# Geldanamycin Derivative Ameliorates High Fat Diet-Induced Renal Failure in Diabetes

**DOI:** 10.1371/journal.pone.0032746

**Published:** 2012-03-06

**Authors:** Hong-Mei Zhang, Howard Dang, Amrita Kamat, Chih-Ko Yeh, Bin-Xian Zhang

**Affiliations:** 1 Department of Clinical Oncology, Xijing Hospital, The Fourth Military Medical University, Xi'an, China; 2 Department of Medicine, Health Science Center, University of Texas, San Antonio, Texas, United States of America; 3 Department of Comprehensive Dentistry, Health Science Center, University of Texas, San Antonio, Texas, United States of America; 4 Audie L. Murphy Division, Geriatric Research, Education and Clinical Center, South Texas Veterans Health Care System, San Antonio, Texas, United States of America; National Cancer Institute, United States of America

## Abstract

Diabetic nephropathy is a serious complication of longstanding diabetes and its pathogenesis remains unclear. Oxidative stress may play a critical role in the pathogenesis and progression of diabetic nephropathy. Our previous studies have demonstrated that polyunsaturated fatty acids (PUFA) induce peroxynitrite generation in primary human kidney mesangial cells and heat shock protein 90β1 (hsp90β1) is indispensable for the PUFA action. Here we investigated the effects of high fat diet (HFD) on kidney function and structure of db/db mice, a widely used rodent model of type 2 diabetes. Our results indicated that HFD dramatically increased the 24 h-urine output and worsened albuminuria in db/db mice. Discontinuation of HFD reversed the exacerbated albuminuria but not the increased urine output. Prolonged HFD feeding resulted in early death of db/db mice, which was associated with oliguria and anuria. Treatment with the geldanamycin derivative, 17-(dimethylaminoehtylamino)-17-demethoxygeldanamycin (17-DMAG), an hsp90 inhibitor, preserved kidney function, and ameliorated glomerular and tubular damage by HFD. 17-DMAG also significantly extended survival of the animals and protected them from the high mortality associated with renal failure. The benefit effect of 17-DMAG on renal function and structure was associated with a decreased level of kidney nitrotyrosine and a diminished kidney mitochondrial Ca^2+^ efflux in HFD-fed db/db mice. These results suggest that hsp90β1 is a potential target for the treatment of nephropathy and renal failure in diabetes.

## Introduction

Diabetic nephropathy is a progressive disorder in diabetic patients and worsens over time. Although hyperglycemia is known as the primary factor underlying the initiation and progression of diabetic nephropathy, the pathogenesis of diabetic nephropathy is complex and remains unclear [1]. Oxidative stress due to increased reactive oxygen species (ROS) production has been postulated to contribute to matrix accumulation, inflammation and tubulointerstitial fibrosis in the diabetic kidney [2]–[4]. Peroxynitrite, formed by the interaction of superoxide and nitric oxide, is a potent oxidant that attacks a variety of biomolecules including proteins, and causes structural and functional damage to tissues and cells. Increased level of nitrotyrosine in proximal tubules of diabetic patients suggest that oxidative injury of the proximal tubules by peroxynitrite may play an important part in the pathogenesis and/or progression of diabetic nephropathy [5]. Improvement of glomerular filtration rate in type 2 diabetes patients with diminished kidney functions by bardoxolone methyl, an agonist of nuclear factor-erythroid 2-related factor 2 that regulates cytoprotective antioxidant pathways, demonstrated the efficacy of antioxidant in treating diabetic nephropathy [6], [7].

Longstanding hyperglycemia, along with other factors, is associated with accelerated decline of kidney function in patients with type 2 diabetic nephropathy [8]. Despite the lack of benefits for all-cause mortality and cardiovascular mortality, intensive hyperglycemic control reduces the risk of diabetic nephropathy and other microvascular complications significantly [9]. In vitro studies also support the critical role of high glucose in regulating matrix protein levels in kidney cells including mesangial [10], endothelial and epithelial cells [11]. Hyperglycemia exposure, albuminuria and other factors interact with the tubular system to cause oxidative stress and interstitial inflammation, which in turn contribute to tubulointerstitial fibrosis and progression of diabetic nephropathy [2], [12]. Exposure of primary human renal proximal tubular cells to high glucose enhances cell proliferation and increases the level of collagen IV and fibronectin [13], [14]. Increased collagen IV expression, mitochondrial dysfunction and excessive ROS generation were observed in murine proximal tubular cells exposed to high glucose [15]–[17].

Despite the critical role of hyperglycemia *per se* in vascular complications of type 2 diabetes, other metabolic factors, such as hyperlipidemia and elevated serum nonesterified fatty acids (NEFA), are clearly involved in the pathogenesis of diabetic nephropathy. Excessive NEFA not only contribute to insulin resistance by various mechanisms [18]–[22], but also cause mitochondrial defects [23]. Our previous studies indicate that polyunsaturated fatty acids (PUFA) induce peroxynitrite generation in various cell types including primary human mesangial cells [24], [25]. The increasing peroxynitrite formation in response to PUFA requires heat shock protein 90β1 (HSP90β1) and is associated with Ca^2+^ efflux from the mitochondria [25], [26]. In the current work, we investigated the role of hsp90 in high fat diet (HFD)-induced renal failure in db/db mice. Our results demonstrated that inhibition of hsp90 with 17-DMAG preserved kidney function, ameliorated glomerular and tubular damage, and improved animal survival in HFD- fed db/db mice. These beneficial effects of 17-DMAG in vivo may result from a reduction of peroxynitrite formation and oxidative damage in the kidney of db/db mice. Our findings provide new insights into molecular mechanisms underlying diabetic nephropathy.

## Results

### High fat diet (HFD) induces decline of kidney function in db/db mice

**Figure 1:**
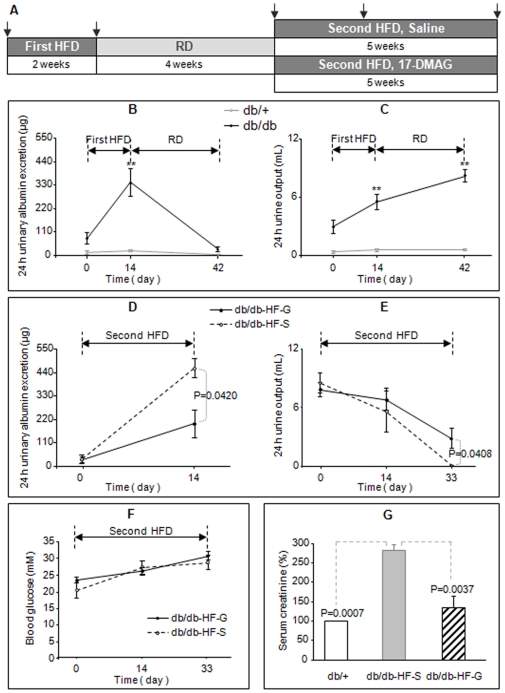
Effects of high fat diet (HFD) and 17-DMAG treatment on kidney function of *db/db* mice. (*A*) Schematic of two phases of HFD feeding and 17-DMAG treatment with the arrows indicating the scheduled kidney function assessments. During the first phase of HFD and the subsequent regular diet (RD) feeding, the 24 h urinary albumin excretion (*B*) and urine output (*C*) were measured to assess kidney functions in *db/db* and the non-diabetic control (*db/+*) mice. During the second phase of HFD feeding, the animals were either injected with saline (*HF-S*) or 17-DMAG (*HF-G*) and the kidney functions were initially assessed by the 24 h urinary albumin excretion (*D*) and urine output (*E*), and then by serum creatinine (*G*) when anuria or oliguria occurred. Blood glucose was measured as indicated in (*F)*. **P<0.01, compared with baseline assessed on day 0 with n = 6–12 per group.

In these experiments, the challenge of db/db mice with HFD was divided into two phases with a four-week regular diet (RD) interval as illustrated in Figure 1A. This design allowed us to test the effect of HFD on kidney function in db/db mice and whether the HFD effects were reversible. Following the first phase of HFD feeding (on HFD for 2 weeks starting at 3 month old), all *db/db* mice showed dramatic increases in urinary albumin excretion and urine output (Figure 1 B and C), rapid bodyweight gain, and elevated blood glucose levels (Figure S1). The urinary albumin excretion (Figure 1 B) and bodyweight (Figure S1) were fully reversed to the pre-HFD treatment levels following discontinuation of HFD for four weeks. However, the increased urine output and blood glucose levels induced by HFD feeding persisted at four weeks following discontinuation of HFD. In parallel control experiments, two-week HFD challenge had no significant effects on urinary albumin excretion and urine output in the non-diabetic heterozygous littermate *db/+* mice (Figure 1 B and C).

### The hsp90 inhibitor 17-DMAG antagonizes HFD-induced decline of kidney functions in db/db mice

Feeding db/db mice with HFD leads to rapid and reversible bodyweight gain because of the increase in fat tissues, which may further exacerbate circulating fatty acids, including PUFA. Linoleic acid (LA), a major component of PUFA in the plasma, interacts with hsp90β1 to cause mitochondrial Ca^2+^ ([Ca^2+^]_m_) efflux and peroxynitrite generation in cell cultures [25], [26]. These pathways may contribute to HFD-induced decline of kidney functions in db/db mice by increasing oxidative stress. In the second phase of HFD challenge (Figure 1), db/db mice were treated with or without 17-DMAG (6.5 µg/kg bodyweight injected intraperitoneally once daily) to test the involvement of hsp90. The dose of 17-DMAG was chosen based on our previous studies showing 17-DMAG at that concentration exerting maximal inhibition on PUFA-induced [Ca^2+^]_m_ efflux but not producing apparent toxicity to cells [26]. In db/db mice without 17-DMAG treatment [injected with saline (vehicle) *db/db-HF-S* group, Figure 1 D], a dramatic increase in urine albumin excretion was observed following two weeks on HFD in the second phase of HFD challenge, mirroring the results in the first phase of HFD feeding (Figure 1B). Interestingly, treatment with 17-DMAG of the db/db mice during the second phase of HFD challenge significantly reduced albuminuria (*db/db-HF-G* group, Figure 1 D).

In contrast to the large increase of 24-h urine volume during two weeks of the first phase of HFD feeding, there was only a small and insignificant decrease of 24-h urine output following initial two weeks on HFD in the second phase (Figure 1E). Interestingly, starting from the 17^th^ day of the second phase of HFD feeding, we observed a dramatic decrease of urine output or development of anuria in saline-injected db/db mice (*db/db-HF-S* group, Figure 1 E), indicating the loss of kidney function and the development of renal failure. Treatment with 17-DMAG resulted in significantly higher 24 h urine output (*db/db-HF-G* group, Figure 1 E), indicating preservation of renal function and prevent of renal failure by 17-DMAG. These beneficial effects of 17-DMAG on kidney functions were observed without measurable changes in blood glucose compared with that of saline-injected animals (*db/db-HF-S vs db/db-HF-G*, Figure 1 F).

The induction of renal failure by prolonged HFD feeding in db/db mice and the beneficial effect of 17-DMAG treatment were further supported by evidence based on serum creatinine measurement. Serum creatinine was determined in animals at the end of four weeks of the second phase HFD feeding. The results indicated that the serum creatinine was 283% higher in saline-injected db/db mice compared to *db/+* mice (*db/db-HF-S vs* db/+, Figure 1G). Interestingly, treatment with 17-DMAG significantly lowered the serum creatinine in HFD-fed db/db mice (*db/db-HF-G vs db/db-HF-S*, Figure 1G) and no significant difference was found between *db/db-HF-G* and *db/+* groups, indicating that 17-DMAG restored mice kidney function to normal creatinine clearance. These data provided evidence that HFD feeding resulted in renal failure in *db/db* mice and 17-DMAG treatment effectively preserved renal functions.

### 17-DMAG mitigates HFD-induced structural damage of glomeruli and tubules

**Figure 2:**
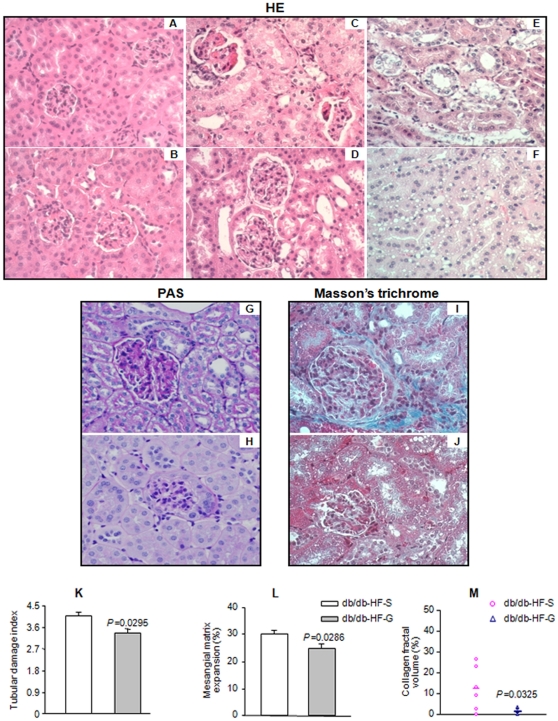
Kidney histopathology of HFD-fed *db/db* mice. Representative figures showing the photomicrographs of HE (*A–F*), PAS (*G*, *H*), and Masson's trichrome stained sections from *db/db-HF-S* group (*C, E, G, I*) and *db/db-HF-G* group (*D, F, H, J*) taken at 200× magnification. Image-based computer assisted analysis was performed to quantify tubular damage index (*K*), mesangial expansion (*L*), and interstitial collagen accumulation (*M*) from 6 animals per group. (A) and (B) showed parallel experiments with HFD-fed *db/+* mice injected with saline and 17-DMAG, respectively.

The effect of HFD and treatment with 17-DMAG on kidney structure was examined with histopathology. The kidney samples were collected at the time of renal failure (as indicated by oliguria, anuria, and mortality) or at the end of the experiments (Day 33 of the second phase HFD feeding after total mortality was observed in saline-injected group of db/db mice) and analyzed by histopathological stains. Inspection of hematoxylin-eosin staining indicated segmental glomerulosclerosis, patches of tubular vacuolation, atrophy and degeneration in saline-injected db/db mice (*db/db-HF-S* group, Figure 2 C and E). These damages induced by HFD in the glomeruli and tubules were largely protected by 17-DMAG (*db/db-HF-G* group, Figure 2 D and F). In parallel experiments, HFD-fed *db/+* mice treated with saline (Figure 2 A) or 17-DMAG (Figure 2 B) did not exhibit any glomerular and tubular abnormalities. Structural damages in the db/db mice kidney was further confirmed by mesangial matrix expansion and tubulointerstitial fibrosis in PAS and Masson's trichrome stained sections (Figure 2 G and I). Interestingly, 17-DMAG treatment effectively ameliorated these abnormalities (Figure 2 H and J). Quantification with morphometric measurements confirmed significant alleviation of tubular damage (Figure 2 K), mesangial matrix expansion (Figure 2 L), and collagen accumulation (Figure 2 M) by 17-DMAG treatment.

### High fat diet causes early death of db/db mice and 17-DMAG treatment improves animal survival

**Figure 3:**
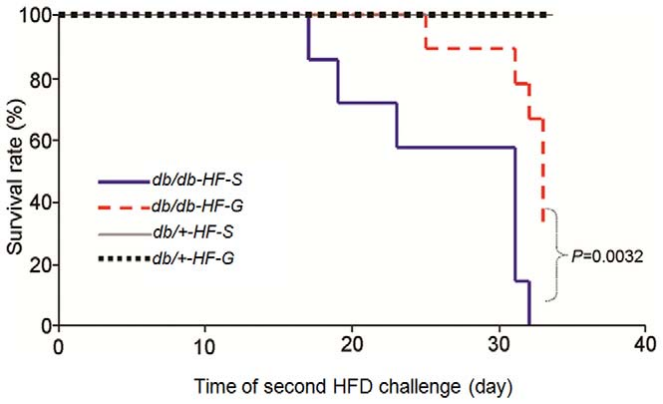
Effect of 17-DMAG on survival rate of HFD-fed *db/db* mice. Kaplan-Meyer survival analysis was performed using the log-rank statistics to measure the difference between the survival curves of d*b/db-HF-S* vs *db/db-HF-G* mice with n = 9 per group. Parallel experiments were performed with *db/+* mice (*db/+-HF-S* and *db/+-HF-G* groups) and no mortality was observed.

Renal failure is expected to cause mortality in the absence of additional treatments such as dialysis or kidney transplantation. Our results indicated that oliguria and anuria in HFD- fed db/db mice were associated with early mortality. As demonstrated in Figure 3, the earliest death among *db/db* mice injected with saline was observed on the 17^th^ day of second phase HFD challenge and complete mortality was observed within 33 days. Treatment with 17-DMAG resulted in a significantly longer survival (*db/db-HF-S* vs *db/db-HF-G*, Figure 3). It is noteworthy that the second phase HFD feeding for 4–5 weeks did not cause renal failure or mortality in *db/+* mice regardless of saline or 17-DMAG treatments (Figure 3).

### 17-DMAG inhibits calcium efflux from the kidney mitochondrial of HFD-fed db/db mice

**Figure 4:**
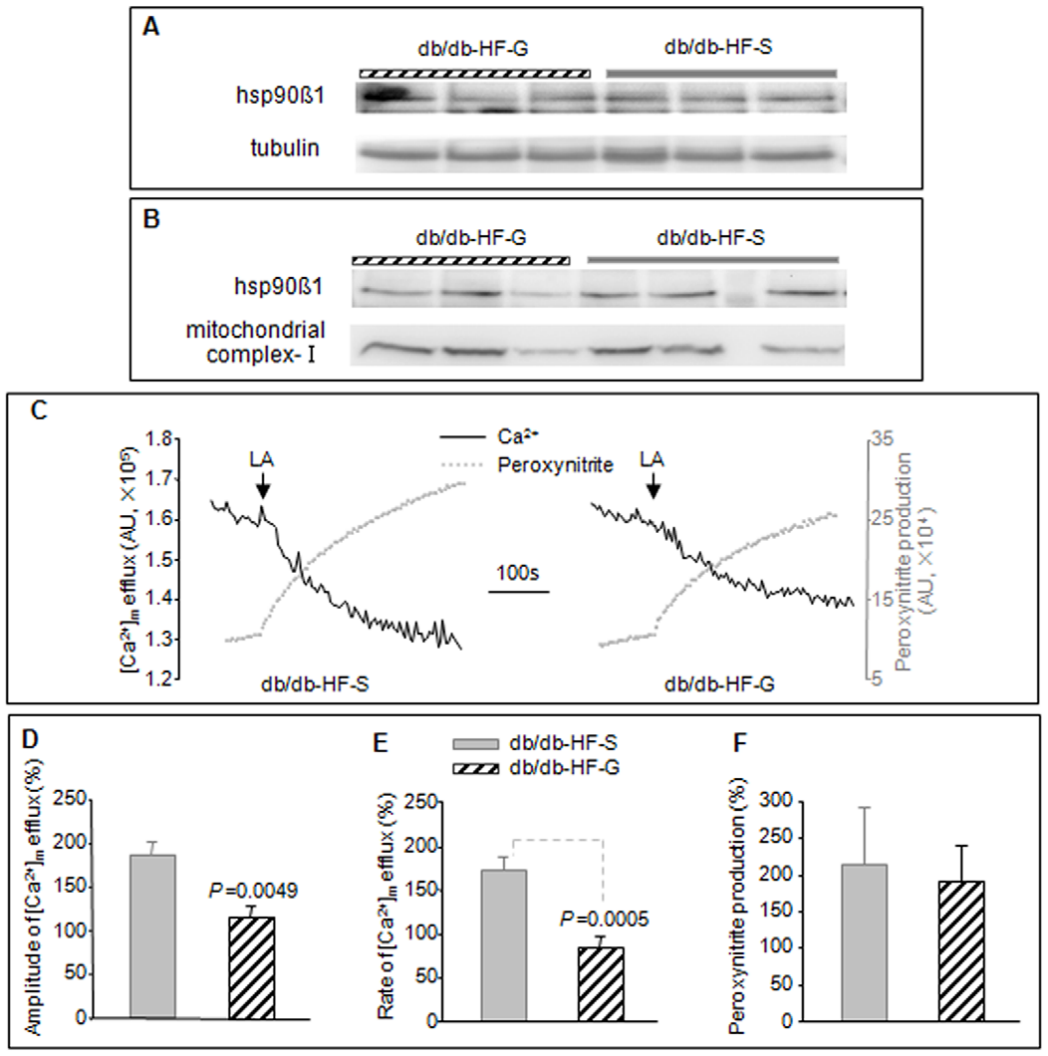
Impact of 17-DMAG on hsp90β1, [Ca^2+^]_m_, and peroxynitrite generation in the kidney. Western blot analysis was performed to assess hsp90β1 in kidney homogenate (*A*) and isolated mitochondria (*B*) of HFD-fed *db/db* mice with 6 animals per group. Linoleic acid (LA)-induced [Ca^2+^]_m_ efflux and peroxynitrite generation (*C–F*) in kidney mitochondria were measured.

Elevated fatty acids, particularly LA and other PUFA, in diabetes may alter mitochondrial functions and ROS generation by [Ca^2+^]_m_ efflux. Treatment of cells with 17-DMAG downregulates hsp90β1 and inhibits LA-induced [Ca^2+^]_m_ efflux [25], [26]. We thus determined whether the beneficial effect of 17-DMAG on HFD-fed db/db mice involved the regulation of the same pathways in the kidney. Hsp90β1 levels in the kidney of HFD-fed *db/db* mice was analyzed by immunoblotting and the results demonstrated similar hsp90β1 levels in the kidney homogenates and isolated mitochondria in animals treated with or without 17-DMAG (*db/db-HF-S* vs *db/db-HF-G*, Figure 4 A and B). Interestingly, as shown in Figure 4 C–E, LA-induced [Ca^2+^]_m_ efflux was significantly diminished in the kidney mitochondria of *db/db* mice treated with 17-DMAG compared to saline. Both the rate and amplitude of LA-induced [Ca^2+^]_m_ efflux were significantly attenuated by 17-DMAG treatment (Figure 4 D and E). The peroxynitrite generation was also attenuated by 17-DMAG in the *db/db-HF-G* group even though the results did not reach statistical significance (Figure 4 F). These data demonstrate that in HFD-fed *db/db* mice, 17-DMAG reduces LA-induced [Ca^2+^]_m_ efflux, which may preserve Ca^2+^-dependent mitochondrial functions and reduce oxidative stress in the kidney.

### 17-DMAG reduces nitrotyrosine level in HFD-fed db/db mice kidney

**Figure 5:**
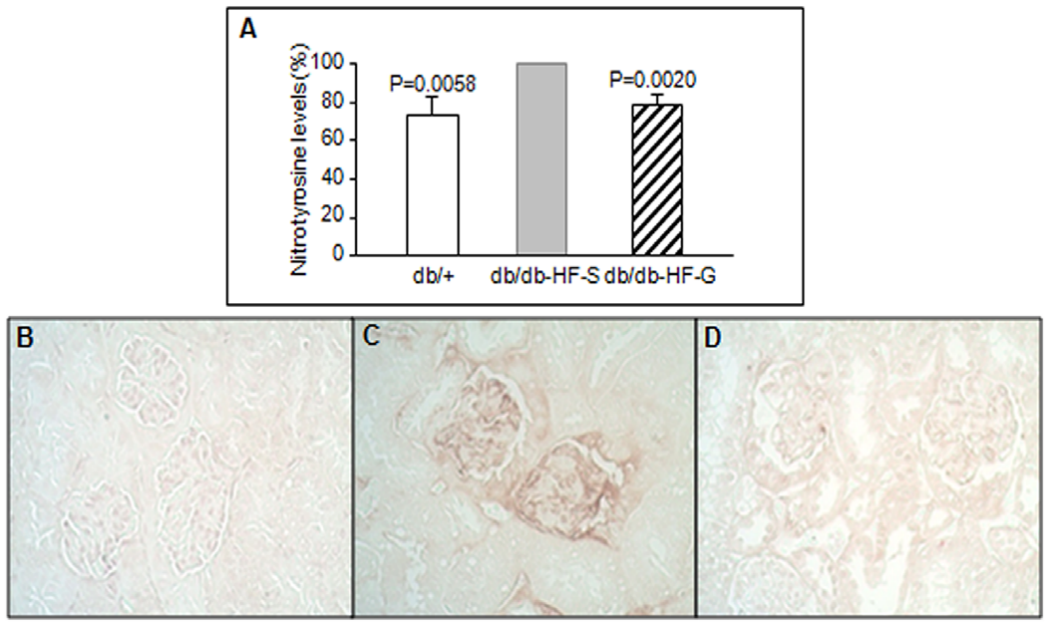
Effect of 17-DMAG on nitrotyrosine levels in the kidney. (A) Western blot analysis of nitrotyrosine was performed with monoclonal antibodies in kidney homogenates. The bar graphs were mean±SE density values from proteins ≤50 kDa normalized to the *db/db-HF-S* group from 6 animals per group. The nitrotyrosine level in kidney sections of *db/+* (C), *db/db-HF-S* (D) and *db/db-HF-G* (E) groups was assessed by immunohistochemistry with same monoclonal antibodies.

Since LA-induced [Ca^2+^]_m_ efflux is coupled to peroxynitrite generation [25] and nitrotyrosine level indicates oxidative damage by peroxynitrite, we measured nitrotyrosine levels in kidney tissues by immunoblotting and immunohistochemistry (Figure 5). As demonstrated in Figure 5 A, nitrotyrosine level in proteins with molecular weight ≤50 kDa was significantly higher in HFD-fed db/db mice injected with saline than in *db/+* mice. Interestingly, 17-DMAG treatment significantly lowered renal tissue nitrotyrosine levels in HFD-fed db/db mice (Figure 5A), indicating a protective effect against nitrosative injury by HFD. The results of immunohistochemistry also indicated high nitrotyrosine levels in HFD-fed db/db mice, particularly in the glomerular region, and 17-DMAG treatment effectively reduced renal tissue nitrotyrosine levels (Figure 5 B–D).

## Discussion

Diabetic renal disease is a complication that develops in a subpopulation of patients with longstanding diabetes [1]. The deterioration in kidney functions including albuminuria and reduction of glomerular filtration rate is associated with histopathological alterations characterized by mesangial matrix expansion, glomerulosclerosis and tubulointerstitial fibrosis [27]. All of these functional and histopathological changes are believed to be the result of an interaction between metabolic abnormality and genetic predisposition [28]. Our results provide evidence that HFD accelerates diabetic nephropathy and caused renal failure in db/db mice, a rodent model of type 2 diabetes (Figures 1, 2, 3). The exacerbated albuminuria but not the 24 h urine output caused by HFD was reversible following discontinuation of HFD (Figure 1). However, prolonged HFD feeding leads to oliguria and anuria, an indication of renal failure, associated with animal death in db/db mice. More severe damage to glomeruli and tubules was also observed in HFD-fed db/db mice (Figure 2). All these deleterious effects of HFD in db/db mice were significantly ameliorated by inhibition of hsp90 with 17-DMAG. Our results suggest that HFD may worsen diabetic nephropathy and cause renal failure by exacerbating the metabolic abnormality in db/db mice.

The levels of plasma glucose and nonesterified fatty acids (NEFA) are elevated in type 2 diabetes and are implicated in diabetic complications. Several clinical studies with large cohorts have shown the role of hyperglycemia as a causative factor in the development and progression of diabetic nephropathy [29]–[31]. Experimental evidence support that hyperglycemia alters the expression of a number of genes involved in matrix protein synthesis and degradation in the diabetic kidney [32]. Nevertheless, hyperglycemia alone is clearly not sufficient to account for the heterogeneity and variability of diabetic nephropathy. We thus used HFD-fed db/db mice to investigate the effect of high lipid ingestion on the progression of diabetic nephropathy. We found that following 2 weeks of HFD feeding, both db/+ and db/db mice exerted excessive accumulation of adipose tissues as indicated by the rapid extra bodyweight gains (11.5% in db/+ and 22% in db/db on HFD, *P*<0.05, n = 5), which were coupled with significantly increased albuminuria in the kidney of db/db mice. Both dietary lipid ingestion and deregulated lipolysis due to insulin resistance may lead to higher circulated NEFA in HFD-fed db/db mice. These observations suggest that the deleterious effect of HFD on the kidney of db/db mice may be due to the exacerbating metabolic abnormality associated with excessive fat accumulation and elevated NEFA.

Kidney mitochondria, the major site of catabolism and oxidation of carbohydrates and lipids, are readily exposed and vulnerable to damaging insults from the exacerbated hyperglycemia and hyperlipidemia in HFD-fed db/db mice and the damage to renal mitochondria may contribute to worsening of nephropathy and renal failure observed (Figures 1 and 2). Morphological and ultrastructural changes of the mitochondria in proximal tubules correlate with deterioration of renal function in diabetes [33], [34]. Increased posttranslational modification of renal mitochondrial proteins through glycation [35], nitration and oxidation [36] is associated with the development of diabetic nephropathy in animal models. Moreover, glycation of mitochondrial proteins is associated with excess superoxide generation [35]. It has been reported that repetitive intraperitoneal injection of NEFA-bond bovine serum albumin leads to functional and structural alterations in mouse kidney with characteristics similar to those of diabetic nephropathy [37]. These renal abnormalities were associated with a decrease in catalase, superoxide dismutase, enzymes involved in NEFA oxidation, and antiapoptotic proteins, and an increase of proinflammatory factors and macrophage infiltration. We noted an imbalance in mitochondrial complex I and III in the renal mitochondria in db/db mice [38], [39]. In cultured cells, linoleic acid and other NEFA has been shown to cause loss of mitochondrial membrane potential, activation of caspase 3, 7, and 9, cytochrome c release and apoptosis, [Ca^2+^]_m_ efflux and peroxynitrite generation [25], [40]. We provided evidence that the last action of NEFA is mediated by hsp90β1 [26]. It is possible that all these NEFA actions upon mitochondria may contribute to the deterioration of diabetic kidney and development of renal failure in HFD-fed db/db mice. The beneficial effect of 17-DMAG on renal function and structure and the counteraction of 17-DMAG upon linoleic acid induced [Ca^2+^]_m_ efflux in renal mitochondria in HFD-fed db/db mice suggest the involvement of these pathways.

As a highly water soluble derivative of geldanamycin, 17-DMAG is an hsp90 inhibitor with potent anticancer activities against a wide range of malignancies. Its application has been expanded to treat a mouse model of spinal and bulbar muscular atrophy [41]. In this study, treatment of *db/db* mice with 17-DMAG during HFD challenge preserves renal function and ameliorates damages to glomeruli and tubules, indicating involvement of hsp90β1 in the pathology of diabetic nephropathy and renal failure. As a chaperone protein, hsp90β1 is abundantly expressed in cells and tissues and is widely distributed in most subcellular organelles, such as plasma membrane, cytosol, endoplasmic reticulum, mitochondrion, and nucleus [26], [42]–[44]. Hsp90 were detected in outer medulla and glomeruli of rat kidney, but no significant alterations were observed in type 1 diabetic rats [45]. Increased hsp90β levels have been observed in muscles of type 2 diabetes patients [46]. Our previous *in vitro* studies have indicated that mitochondrial hsp90β1 is involved in regulating cytosolic Ca^2+^ and [Ca^2+^]_m_ homeostasis [24]–[26]. A major role of [Ca^2+^]_m_ is to stimulate oxidative phosphorylation by activation of multiple dehydrogenases [47] and ATP synthesis [48]. [Ca^2+^]_m_ inhibits the generation of ROS from complexes I and III under normal conditions whereas its overload promotes ROS generation and apoptosis [49]. The enhanced [Ca^2+^]_m_ efflux in renal mitochondria of HFD-fed *db/db* mice may diminish the inhibitory effect of [Ca^2+^]_m_ on ROS production and thus leads to overproduction of superoxide and peroxynitrite. Furthermore, elevated NEFA, particularly linoleic acid, may augment the interaction with hsp90β1 and [Ca^2+^]_m_ efflux to deplete [Ca^2+^]_m_ and overproduce peroxynitrite, which exaggerates kidney oxidative/nitrosative injuries and eventuates renal failure. This paradigm is supported by the facts that enhanced [Ca^2+^]_m_ efflux and nitrotyrosine levels were found in the kidney of HFD-fed *db/db* mice and inhibition of hsp90 with 17-DMAG ameliorated these HFD effects (Figures 4 and 5). The significant elevation of plasma linoleic acids and arachidonic acids [50], [51] and kidney nitrotyrosine levels [5], [52], [53] in type 2 diabetes patients indicate that a similar scenario may also occur in human beings.

Although 17-DMAG effectively reduced [Ca^2+^]_m_ efflux and nitrotyrosine levels in the kidney of HFD-fed *db/db* mice, we did not observe a significant reduction in the peroxynitrite generation coupled to linoleic acid-induced [Ca^2+^]_m_ efflux (Figures 4 and 5). The reason for the different responses to hsp90 inhibition with 17-DMAG on peroxynitrite generation in mitochondria from *db/db* mice and cultured human mesangial cells [25] is currently unclear. Whether mitochondria in different cell types of kidney (predominantly epithelial cells with few other cell types including mesangial cells) respond to 17-DMAG distinctly requires further investigations.

In summary, our results indicate that HFD aggravates nephropathy and causes renal failure in diabetes and these effects of HFD are antagonized by 17-DMAG. Decreased [Ca^2+^]_m_ efflux and lowered nitrotyrosine levels may account for the beneficial effect of 17-DMAG in diminishing nitrosative injury, preserving kidney function and structure, and improving animal survival. These results suggest that hsp90β1 is a potential target for prevention or treatment of nephropathy and renal failure in diabetes. 17-DMAG or other hsp90 inhibitors might represent new and promising therapeutic candidates for diabetic nephropathy and renal failure.

## Materials and Methods

### Animals

The animal protocols were approved by the Institutional Animal Care and Use Committee, South Texas Veterans Health Care System. Male *db/db* (BKS.*Cg-m+/+Lepr^db^*/J), age and sex matched *db/+* mice were acquired from Jackson Laboratories at age of 10 weeks and housed 4/cage or less. Animals were habitat for 2 weeks in a temperature- and humidity-controlled facility with a 12:12-h light-dark cycle, fed ad libitum with regular diet (7012 Teklad LM-485, Harlan Laboratories) and had free access to water. The mice were challenged with high fat diet (HFD) (TD.06414, Harlan Laboratories, 60.3% calories from fat) starting at 12 weeks of age as depicted in Figure 1A. The HFD challenge was divided into two phases: in the first phase the mice were fed with HFD for 2 weeks followed by a 4-week regular diet period. The second phase of HFD feeding was started after the 4-week regular diet interval and continued for 5 weeks because of a total mortality in HFD-fed db/db mice. At the beginning of the second HFD feeding, *db/db* mice were randomly assigned to 2 groups and intraperitoneally injected with either saline (vehicle, *db/db-HF-S*) or 17-DMAG (6.5 µg/kg bodyweight, *db/db-HF-G*; InvivoGen). Bodyweight of the animals was followed weekly and the dose of 17-DMAG was adjusted according to bodyweight gains.

### Assessment of renal function by 24-h urine output and urinary albumin or serum creatinine

Urine collection and other physical parameters were measured following the schedule in Figure 1A. The animals were fasted for 6 h (9:00 AM-3:00 PM) prior to blood glucose measurement with a glucometer (Accu-Chek, Roche). 24 h urine samples were collected from individual mice housed in metabolic cages and the total urine volume was measured to index urine output. Urinary albumin concentrations were determined using a murine albumin ELISA kit (Albuwell M Kit; Exocell, Philadelphia, PA). In mice with oliguria or anuria, serum creatinine concentrations were determined by the Creatinine Companion kit (Exocell) to indicate renal functions.

### Histopathology

Formalin-fixed, paraffin-embedded kidney sections were stained with haematoxylin and eosin (HE), periodic acid-Schiff (PAS), or Masson's trichrome and analyzed to evaluate kidney damages in a blinded manner. The area of glomerular PAS staining was measured by image analysis using Image-Pro Plus 4.5 (Media Cybernetics, Silverspring, MD) as described [54]. A semi-quantitative assessment was performed [55] in HE-stained slides to evaluate the extent of tubular damage and graded from 1 to 5 as follows: 1: vacuolation of cytoplasm in <20% of tubules; 2: vacuoles in 20% to 40% of tubules; 3: vacuoles in 40% to 60% tubules with minimal distortion of tubular structures; 4: vacuoles in 60% to 80% of tubules with large and marked distortion of tubular profiles, pyknotic nuclei, patches of tubular atrophy and tubular degeneration; and 5: >80% of tubules with severe vacuolation, or tubular atrophy and degeneration. The fractal collagen volume was assessed by point counter grid using ImageJ (NIH) program to quantify the blue stain in the trichrome-stained sections [56].

### Measurement of [Ca^2+^]_m_ and peroxynitrite in mitochondria

Kidney mitochondria were prepared from mice and LA-induced [Ca^2+^]_m_ efflux and peroxynitrite generation were assessed as previously described [25], [26]. Briefly, fresh kidney tissues (0.1–0.2 g) were homogenized in 5–10 ml MB1 solution containing 250 mM mannitol, 75 mM succinic acid, 0.1 mM EDTA, 0.5 mM EGTA, 10 mM HEPES, pH 7.4 at 4°C. The homogenates were centrifuged at 329 g for 15 min and mitochondria in the supernatant were collected by further centrifugation at 10000 g for 30 min. Mitochondria were double labeled with X-rhod-1 AM (2 µM) and 2′,7′-dichlorodihydrofluorescein diacetate (1 µM) at 37°C for 60 min and LA-induced [Ca^2+^]_m_ efflux and peroxynitrite generation were measured by changes in X-rhod-1 and 2′,7′-dichlorodihydrofluorescein fluorescence, respectively [25].

### Assessment of hsp90β1 and nitrotyrosine

Immunoblotting and immunochemistry were performed to detect hsp90β1 and nitrotyrosine levels. In immunoblotting experiments, total and mitochondrial protein were isolated from mice kidney as previously described [25]. The primary antibodies used were as follows: mAb anti-mouse nitrotyrosine (1∶1000, clone 1A6, Millipore); pAb anti-mouse GRP94 (1∶1000, Santa Cruz Biotechnology), mAb anti-mitochondrial complex 1 NDUFS-3 subunit (1∶2000, Invitrogen) and mAb anti-α-tubulin (1∶1000, Invitrogen). The secondary antibodies were HRP-linked anti-rabbit or anti-mouse IgG (1∶5000, GE Healthcare UK Limited). For the immunochemistry staining with mouse mAb, a specific procedure was performed as previously described [57] to eliminate the direct interaction between antigen and secondary antibody. Prior to application to the specimen, the primary mAb was incubated with secondary biotinylated anti-mouse immunoglobulin, resulting in the binding of biotinylated secondary antibody to the primary mAb. Normal mouse serum was added to the mixture to bind the residual biotinylated anti-mouse immunoglobulin, preventing the potential interaction with endogenous immunoglobulin in the specimen. The nitrotyrosine levels were detected by streptavidine-peroxidase (Dako ARK Kit) and peroxidase substrate solution (DAB Substrate Kit, Vector Laboratories).

### Statistical analysis

Results presented as mean±S.E and Student's t-test was used to evaluate the differences between two groups. Kaplan-Meyer survival analysis was performed using the log-rank statistic to test for a significant difference among the survival curves. Differences were considered statistically significant at *P*<0.05.

## Supporting Information

Figure S1 **Effects of HFD and 17-DMAG treatment on bodyweight and blood glucose levels of db/db mice.** HFD caused rapid gains in bodyweight that was reversed to the baseline values measured prior to HFD feeding following four weeks discontinuation of HFD. The elevation of blood glucose after first HF seemed irreversible even after four weeks on RD feeding. The bodyweight gain was significantly potentiated in *db/db-HF-D* group compared to *db/db-HF-S* group (Figure S1). The persistently elevated blood glucose level that were not reversed during the four week interval of RD did not show further significant increases in the second HFD challenge (Figure S1, right panels). *P<0.05, **P<0.001, compared to baseline values assessed on day 0 from 6–12 animals per group.
(DOCX)
