# Supplementary material for: Geldanamycin Derivative Ameliorates High Fat Diet-Induced Renal Failure in Diabetes
Source: PLoS One. 2012 Mar 6;7(3):e32746. doi: 10.1371/journal.pone.0032746 (PMC3295767; doi:10.1371/journal.pone.0032746)
Supplement: Figure S1 — Effects of HFD and 17-DMAG treatment on bodyweight and blood glucose levels of db/db mice. HFD caused rapid gains in bodyweight that was reversed to the baseline values measured prior to HFD feeding following four weeks discontinuation of HFD. The elevation of blood glucose after first HF seemed irreversible even after four weeks on RD feeding. The bodyweight gain was significantly potentiated in db/db-HF-D group compared to db/db-HF-S group (Figure S1). The persistently elevated blood glucose level that were not reversed during the four week interval of RD did not show further significant increases in the second HFD challenge (Figure S1, right panels). *P<0.05, **P<0.001, compared to baseline values assessed on day 0 from 6–12 animals per group. (DOCX) [file pone.0032746.s001.docx]

**Supplementary information:**

**Figure S1.** Effects of HFD and 17-DMAG treatment on bodyweight and blood glucose levels of db/db mice


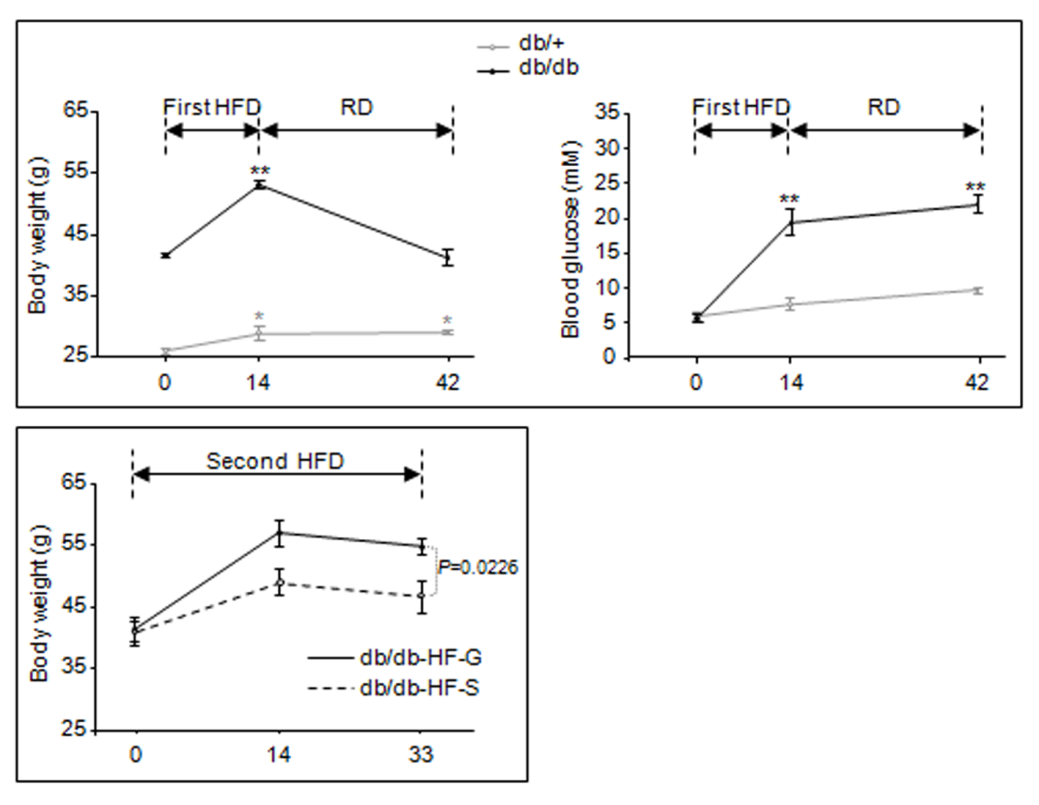


**Supporting Information Legends**

HFD caused rapid gains in bodyweight that was reversed to the baseline values measured prior to HFD feeding following four weeks discontinuation of HFD. The elevation of blood glucose after first HF seemed irreversible even after four weeks on RD feeding. The bodyweight gain was significantly potentiated in *db/db-HF-D* group compared to *db/db-HF-S* group (supplemental Figure S1). The persistently elevated blood glucose level that were not reversed during the four week interval of RD did not show further significant increases in the second HFD challenge (supplemental Figure S1, right panels). **P*<0.05, ***P*<0.001, compared to baseline values assessed on day 0 from 6-12 animals per group.
